# Effect of Yttrium doping on antibacterial and antioxidant property of LaTiO_3_

**DOI:** 10.1186/s11671-023-03942-1

**Published:** 2023-12-18

**Authors:** V. G. Prabitha, Jhelai Sahadevan, Maya Madhavan, S. Esakki Muthu, Ikhyun Kim, T. K. Sudheer, P. Sivaprakash

**Affiliations:** 1 Department of Physics, Government College for Women, Thiruvananthapuram, Kerala 695014 India; 2 Centre for Biophotonics and Technology, Karpagam Academy of Higher Education, Coimbatore, Tamil Nadu 641021 India; 3 Department of Biochemistry, Government College for Women, Thiruvananthapuram, Kerala 695014 India; 4 Department of Physics, Centre for Materials Science, Karpagam Academy of Higher Education, Coimbatore, Tamil Nadu 641021 India; 5 Department of Mechanical Engineering, Keimyung University, Daegu, 42601 Republic of Korea

**Keywords:** Auto combustion, Gram-negative strain, Gram-positive strain, *E. coli*-443, *Staphylococcus aureus*-902

## Abstract

The advancement of multidrug-resistant bacterial strains and their adverse effects is one of the most significant global health issues. The perovskite nanomaterial with combined antioxidant and antibacterial activities in one molecule has the potential for improved therapeutic solutions. In this work, Yttrium-doped Lanthanum Titanate (LaTi_1 −*x*_Y_*x*_O_3_, where *x* = 0, 0.05, and 0.1) was synthesized using auto combustion technique. Excellent crystalline structure with a tetragonal system is revealed by X-ray diffraction analysis (XRD). UV–Visible diffuse reflectance spectroscopy (UV–Vis DRS), Fourier transform infrared (FTIR), and photoluminescence (PL) were used to study its optical characteristics. The field emission scanning electron microscope (FE-SEM) shows rod-like pellet-shaped Yttrium-doped nanostructures, and the elements present were confirmed with the Energy Dispersive X-Ray Analysis (EDAX). Various concentrations of the synthesized materials were tested for antibacterial activity against Gram-positive (*Staphylococcus aureus* 902) and Gram-negative (*E. coli* 443) strains using the agar-well diffusion method with gentamicin antibiotic as a positive control. High antibacterial activity of 87.1% and 83.3% was shown by 10% Yttrium-doped LaTiO_3_ (LY(0.1)TO) at 500 mg/mL against both positive and negative stains, respectively. Moreover, the antioxidant properties of synthesized materials were assessed with IC50 values of 352.33 µg/mL, 458.055 µg/mL, and 440.163 µg/mL for samples LaTi_1 −* x*_Y_*x*_O_3_, where x = 0, 0.05, and 0.1 respectively. The antibacterial and antioxidant capabilities of the proposed samples illustrate their applicability in various biomedical applications.

## Introduction

Antibiotic resistance is considered a pressing global health hazard, with implications that not only go beyond higher mortality and health complication risks but also increased healthcare expenses [1, 2]. Although, development of novel antibiotics is the sole practical remedy for addressing this issue, it takes years for the development of new antibiotics. Nanoparticles (NPs) that can overcome antibacterial resistance have recently emerged as powerful tools to combat the emerging deadly bacterial infections [3]. The large surface area to volume ratio of the nanoparticles, which is vitally important for microbial attachment and effective penetration into cell, make them an effective platform for the delivery of therapeutics with minimal side effects. Various investigations show that metal and metal oxides nanoparticles such as Silver (Ag), silver oxide (Ag_2_O), titanium dioxide (TiO_2_), silicon (Si), copper oxide (CuO), zinc oxide (ZnO), gold (Au), calcium oxide (CaO) and magnesium oxide (MgO) exhibit antimicrobial activity [4–6]. Perovskite nanostructures with tuneable optical properties have recently garnered much attention. The antibacterial properties of perovskite against different bacterial strains were investigated by Yang et al. [7]. Akbari et al. carried out a comparative study to explore the antibacterial activity of ZnO and CsPbBr_3_ towards *Escherichia coli* [8]. Experimental outcomes showed that the antibacterial effect of perovskite is better than that of metal oxide nanoparticles. In another study, lanthanum aluminate nanoparticles were shown to display maximum antibacterial effect for Gram-negative bacteria Pseudomonas aeruginosa due to the interaction between the positively charged nanoparticle and the negatively charged cell wall [9]. Better antibacterial activity of LaNiO_3_ against Gram-positive bacteria, *Staphylococcus aureus* was reported by Jadhav et al. [10]. Among the different perovskites, lanthanum titanates (LaTiO_3-δ_) are promising materials with numerous applications in different areas such as electrooptical, piezoelectric, dielectric, ferroelectric, and electrical conductivity, because of their unique dielectric, electrical conductivity, ferroelectric, and catalytic properties [11–13]. Yet, there is not much scientific literature available regarding the biological activity studies of lanthanum titanates.

Along with the antibacterial activity, the antioxidant property is also needed for efficient therapeutic applications of the same nanomaterial. Oxidative stress caused by reactive oxygen species (ROS) is a factor proven to be associated with many diseases. Controlling ROS levels by employing antioxidants is one of the most effective therapeutic modalities for the management of neurological disorders and cardiovascular diseases [14]. Liu mentions the new area of antioxidative nanomaterials, which have several benefits over traditional antioxidants, including stability and the ability to scavenge numerous free radicals [15]. Vindhya reports Nickel-doped CuO nanoparticles have higher antioxidant properties as well as antibacterial activity than pure CuO nanoparticles [16]. It is imperative to investigate the antioxidant capacities of perovskite materials along with their antibacterial properties.

Doping nanoparticles offers a versatile means of tuning the properties without altering their large surface area, so that their performance can be enhanced. Over the past few decades, there has been a lot of research done on perovskite-type oxides, particularly those having rare earth ions like La on the A site and transition-metal ions on the B site [13]. Doping in the cationic sites can significantly enhance the materials' electrical, magnetic, optical, and chemical properties. Singh et al. [17] reports that the doping of potassium in LaFeO_3_ has a positive impact on electron donor characteristics and surface mobility, which enhances the antibacterial activity. There is a dearth of research in the literature on the antibacterial and antioxidant properties of A/B site-doped lanthanum titanates, which calls for greater investigation.

In this study, Yttrium is used as a dopant, as the inclusion of yttrium in titanium is known to cause a noticeable reduction in grain growth and significant grain refinement in both the prior α and β-grain sizes [18, 19]. It was found in the literature that only a few antibacterial studies were carried out with B site doped perovskites. Here, Yttrium-doped in the B site of LaTiO_3_ perovskite prepared using auto combustion method and investigated for its antibacterial, and antioxidant properties.

## Materials and methods

### Materials

Yttrium doped Lanthanum titanate LaTi_1 − *x*_Y_*x*_O_3_ (where  *x *= 0, 0.05 and 0.1) powders were prepared by auto combustion followed by calcination. The samples were named as LTO, LY(0.05)TO, LY(0.1)TO respectively for x = 0, 0.05 and 0.1 concentrations. Lanthanum (III) Nitrate hexahydrate (La(NO_3_)_3_.6H_2_O) [Merck, Germany, 99.999%], Titanium Nitride (TiN) [Merck, Germany, 99.998%]_,_ Yttrium (III) Nitrate hexahydrate (Y(NO_3_)_3_·6H_2_O) [Merck, Germany, 99.99%], Ammonia solution 25% (NH_4_OH) [Emplura, Merck], NaOH [Merck, Mumbai, 97%] and DI water [aqua] are the precursor used to synthesis the materials. The chemicals are all reagent-grade, have a high purity (99.9%), and are ready to use right out of the box.

### Synthesis—Auto combustion method

**Fig. 1:**
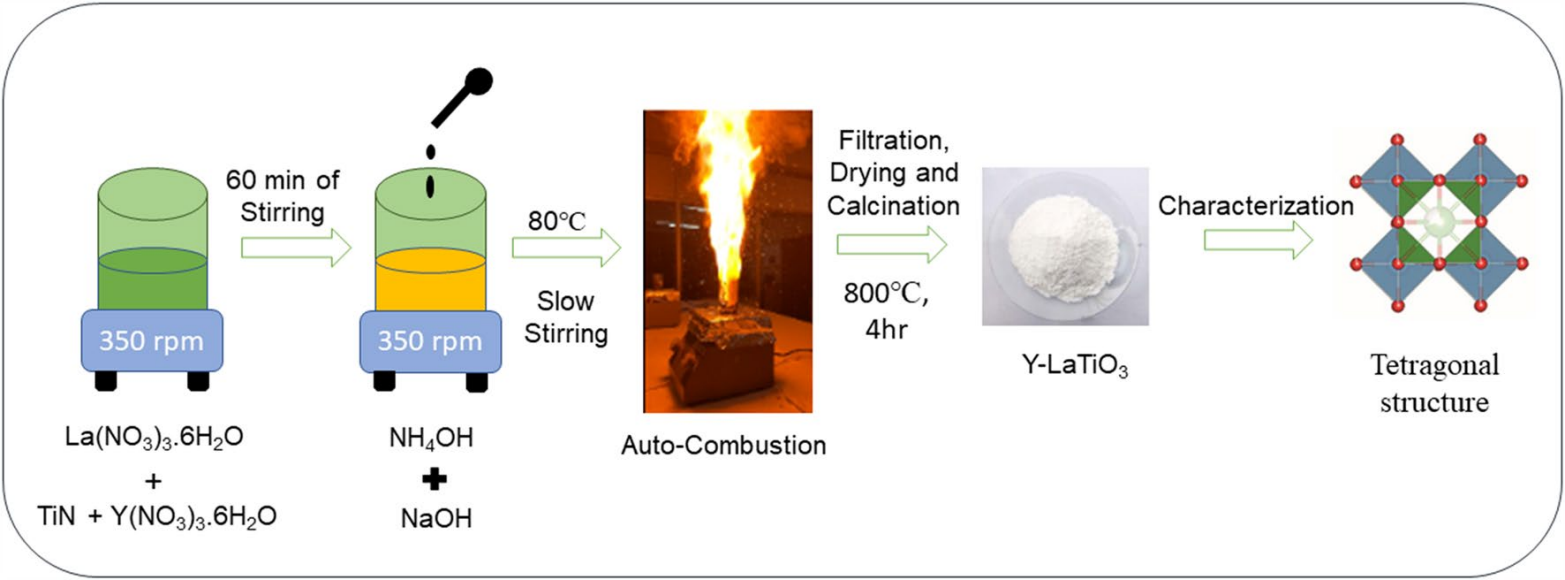
Systematic diagram showing the synthesis process

The synthesis process is systematically shown in Fig. 1. To generate a homogenous mixture, 1 mol (M) of La(NO_3_)_3_.6H_2_O, ‘(1 − *x*)’ M of TiN, and ‘*x*’ M of Y(NO_3_)_3_·6H_2_O (where *x* = 0, 0.05 and 0.1) were dissolved in DI water (80 mL) under continuous stirring for 60 min. To hydrolyse the mixture, 3.5 ml of NH_4_OH solution was then added dropwise. The solution was then treated with 1 M of NaOH until a pH of 11 was achieved. NaOH is utilised as a hydrolysing agent and to boost the deprotonation of ammonia. The above solution was heated to 80 ºC with continuous steering until solution becomes gel. Then the steering slows down before combustion process take place. After combustion of the samples, we naturally cool the sample and bringing the final product down to room temperature (RT), it was then mixed with DI water. The final product was repeatedly centrifuged with DI water, ethanol, and acetone to separate the unreacted contaminants after it had cooled to room temperature. The precipitate was then filtered and calcined for 4 h at 800 °C in an ambient atmosphere. The whole synthesis process is shown in Fig. 1.

### Characterization technique

The analysis of the crystalline structure and phase of the catalysts in their as-prepared state was conducted using powder X-ray diffraction. The instrument employed for this analysis was the Empyrean, Malvern Panalytical. Cu Kα radiation with a wavelength of 1.5418 Å was utilised for the X-ray diffraction measurements. The morphology of the sample was examined using field emission scanning electron microscopy (FESEM) with the Zeiss (SIGMA), Bruker. The Fourier transform infrared (FT-IR) spectra were conducted using a Thermo Fisher Scientific instrument. To examine the optical responses and calculate the band gap, the synthesised samples were analysed using UV–Vis diffuse reflectance spectroscopy (Jasco). The energy associated with the optical band gap of the nanoparticles was determined by employing Tauc's relation, which relies on the analysis of the absorption spectra of such nanoparticles. The equation can be rewritten as (αhν)^n^ = A(hν − E_g_), where hv represents the energy of a photon, A is a constant, and in the given context, α represents the coefficient of optical absorption, hv denotes the energy of a photon E_g_ (for n = 1/2) signifies a direct bandgap, and A represents a constant that varies with energy. The Photoluminescence (PL) spectroscopy was analysed using Horiba.

### Antibacterial study

The agar-well diffusion method was utilised to quantify the antibacterial activity. The medium was made by dissolving 2.8 g of Nutrient Agar Medium (HI Media), which is commercially available, in 100 mL of distilled water. The solution was subjected to autoclaving at a pressure of 15 pounds per square inch (psi) and a temperature of 121 °C for 15 min. The medium that had undergone autoclaving was thoroughly mixed and thereafter poured onto petri plates with a diameter of 100 mm and a volume of 25–30 mL per plate while it was still in a molten state. The nutrient broth was made by dissolving 2.8 g of a commercially available nutrient medium (HiMedia) in 100 millilitres of distilled water and subsequently heating it to its boiling point to ensure complete dissolution of the medium. The medium was administered as required and subjected to sterilisation using autoclaving at a pressure of 15 pounds per square inch (121 °C) for a duration of 15 min. Petri plates were prepared by adding 20 mL of nutrient agar medium. These plates were then inoculated with bacterial strains that had been cultured for 24 h. The optical density (OD) of the bacterial cultures was corrected to a value of 0.5 using the McFarland standard. The bacterial strains used in this experiment were *Staphylococcus aureus* (strain 902) and *Escherichia coli* (strain 443). The wells were incised, and various concentrations of LTO, LY(0.05)TO, and LY(0.1)TO samples (500, 250, 100, and 50 µg/mL) were introduced. Subsequently, the plates were subjected to incubation at a temperature of 37 °C for 24 h. The measurement of the diameter of the inhibitory zone generated around the wells was used to assess the antibacterial activity. A positive control was employed in the form of a gentamicin antibiotic. The numbers were computed with Graph Pad Prism 6.0 software, developed in the United States.

### Antioxidant study

Chang et al., used the DPPH test to determine the radical scavenging activity. A 0.1 millimolar (mM) solution of 2, 2-diphenyl-1-picrylhydrazyl (DPPH) was generated by dissolving 4 mg (mg) of DPPH in 100 mL of methanol. Various concentrations of the sample, including 12.5, 25, 50, 100, and 200 µg/mL, were prepared by diluting the stock solution to a final volume of 20 µL using DMSO. Subsequently, 1.48 ml of a DPPH solution with a concentration of 0.1 mM was added. The reaction mixture is subjected to incubation under light-restricted conditions at ambient temperature for a duration of 20 min. The absorbance of the combination was measured at a wavelength of 517 nm after a duration of 20 min [20]. A control sample consisting of 3 ml of DPPH was used. The IC50 value of the sample was determined using the ED 50 PLUS V1.0 Software. The assessment of free radical scavenging ability was quantified as the percentage of inhibition, calculated by employing the following formula: Percentage of inhibition = ((Pc − Ps)/Pc) × 100, where Pc represents the absorbance value of the control and Ps represents the absorbance value of the sample.

## Result and discussion

**Fig. 2:**
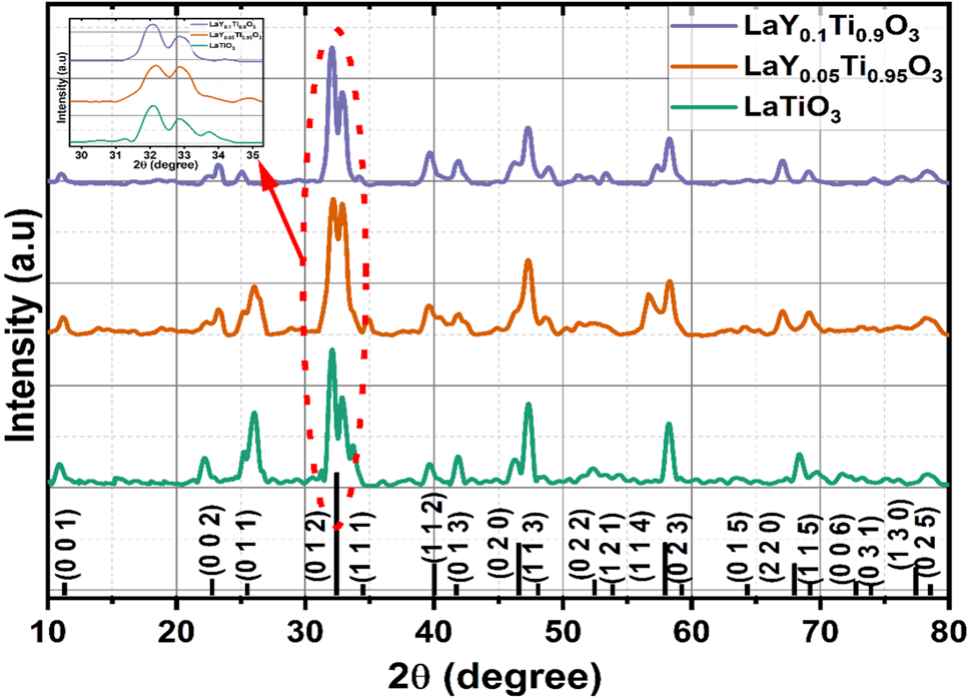
X-ray diffraction patterns of LaY_*x*_Ti_1 − *x*_O_3_ (*x* = 0, 0.05 and 0.1)

Figure 2 depicts the powder X-ray diffraction patterns of LaY_*x*_Ti_1 − *x*_O_3_ (*x* = 0, 0.05 and 0.1) samples calcinated at 800 °C for 4 h. The LYTO samples consisted entirely of a single phase and shows a tetragonal crystal structure with the space group of p 4/mmm (123), which is confirmed by ICSD No: 77674 and its polycrystalline planes were marked in the figure. The crystalline size for the primary peak is determined to be 19, 13 and 12 nm, whereas the average crystalline size is calculated to be 16 nm, 11 nm and 10 nm for LaY_*x*_Ti_1 − *x*_O_3_ (*x* = 0, 0.05 and 0.1) samples respectively using the Debye–Scherrer (1.1) equation.

1.1 $$D=\frac{k\lambda }{\beta cos\theta }$$

Regarding the Scherrer equation for determining crystallite size, D stands for crystallite size, k for the Scherrer constant, which is usually taken to be 0.9, λ for the wavelength of X-ray radiation (0.15418 nm for Cu Kα), θ for the Bragg angle, and β for the FWHM of the diffraction peak measured at 2θ in radians. The increase in yttrium concentration in the Ti site reduces the crystalline size. The LTO nanoparticles can penetrate through the bacterial walls and cause further damage to cell due its low crystalline size. Table 1 shows the percentage variation of unit cell parameters compared with ICSD standard data card no 77674 calculated using the X’pert high score plus. The calculated lattice parameter values are a = b = 3.8872 Å and c = 7.7360 Å, volume of cell is 116.8935 Å^3^, when compared with standard data (ICSD collection code: 77674) it has reduced lattice parameter a = b by 0.277%, c by 0.7569% and its volume by 1.31%. The crystallinity of LaY_*x*_Ti_1 − *x*_O_3_ (x = 0, 0.05 and 0.1) samples are calculated and the values are 39.93%, 41.57% and 57.27% respectively. This shows that the yttrium on titanium site increases the crystallinity of the sample. Also, the main peak (0 1 2) shifts towards lower angle which is due to the substitution of large ionic radii (104 pm) of yttrium into the small ionic radii of Ti (68 pm).

**Table 1:** Lattice parameter of LTO NPs

Unit cell parameters	Standard value (Å) ICSD No: 77674	Calculated value (Å)	Percentage difference (%)
a (Å)	3.8980	3.8872	− 0.277
b (Å)	3.8980	3.8872	− 0.277
c (Å)	7.7950	7.7360	− 0.75696
Unit cell volume (Å^3^)	118.44	116.8935	− 1.31

**Fig. 3:**
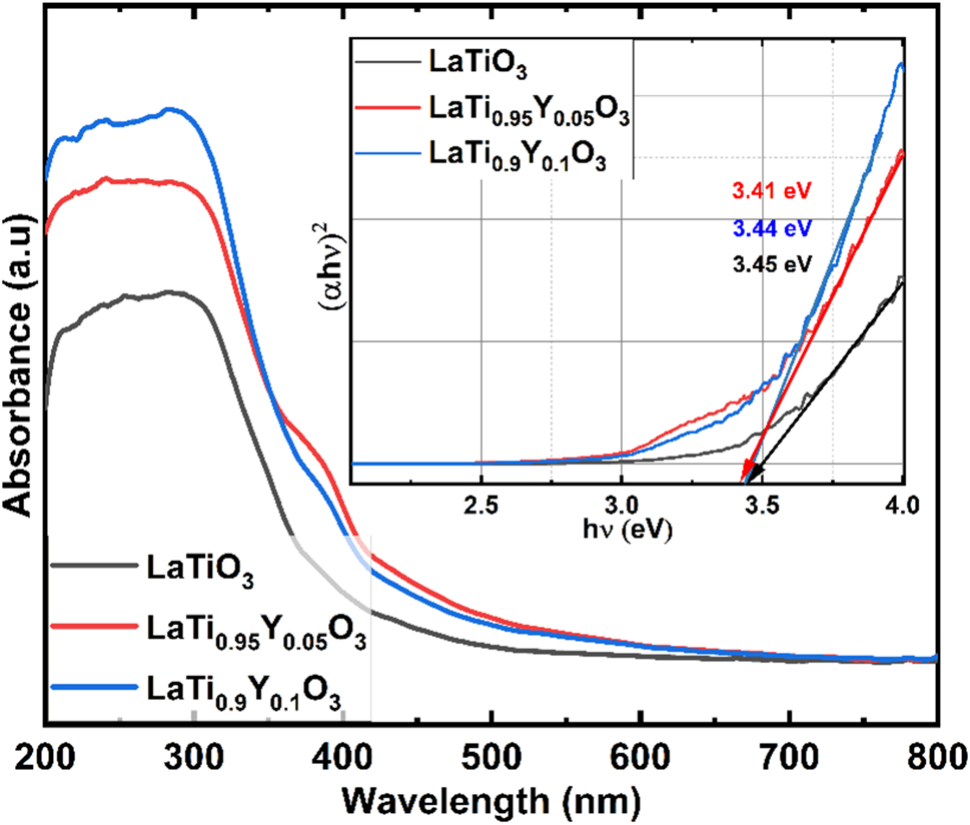
UV-DRS absorption spectrum of LaY_*x*_Ti_1 − *x*_O_3_ (*x* = 0, 0.05 and 0.1) from 200 to 800 nm

The optical characteristics and band gaps of LaY_*x*_Ti_1 − *x*_O_3_ (x = 0, 0.05 and 0.1) were examined using UV–visible diffuse reflectance spectroscopy as shown in Fig. 3.The result revealed a notable shift in absorption towards a higher wavelength range as the concentration of dopant Y increased within the LTO lattice. The broad optical absorption characteristics observed in the rare earth-doped LTO are attributed to the transitions occurring between the O 2*p* state and the Ti 3*d*/4*f* states. [21].Typically, LaTiO_3_ exhibits a band gap value of 3 eV, as reported in reference [22], positioning it as an indirect band gap material. However, there exists a variance between the experimentally determined and calculated band gap values for LTO. As the concentration of Y in LaTiO_3_ increases, the band gap values systematically decrease. This reduction can be attributed to the structural disorder attributed from vacancies in the La lattice, oxygen vacancies, and the distortion of TiO_6_ [23]. The presence of structural defects results in the creation of localized states within the band gap. This inhomogeneous distribution of charge caused by these defects leads to the entrapment of electrons, thereby influencing and altering the relationship within the band gap values [24]. The optical band gap energies (E_g_) were calculated using Tauc relation for LaY_*x*_Ti_1 − *x*_O_3_ (*x* = 0, 0.05 and 0.1) are 3.45, 3.41, and 3.44 eV, respectively, by analysing the intercepts of the tangents in the inset of Fig. 3. Thus, the interaction of Y ions with LTO lattice was verified by the reduced band gap values.

**Fig. 4:**
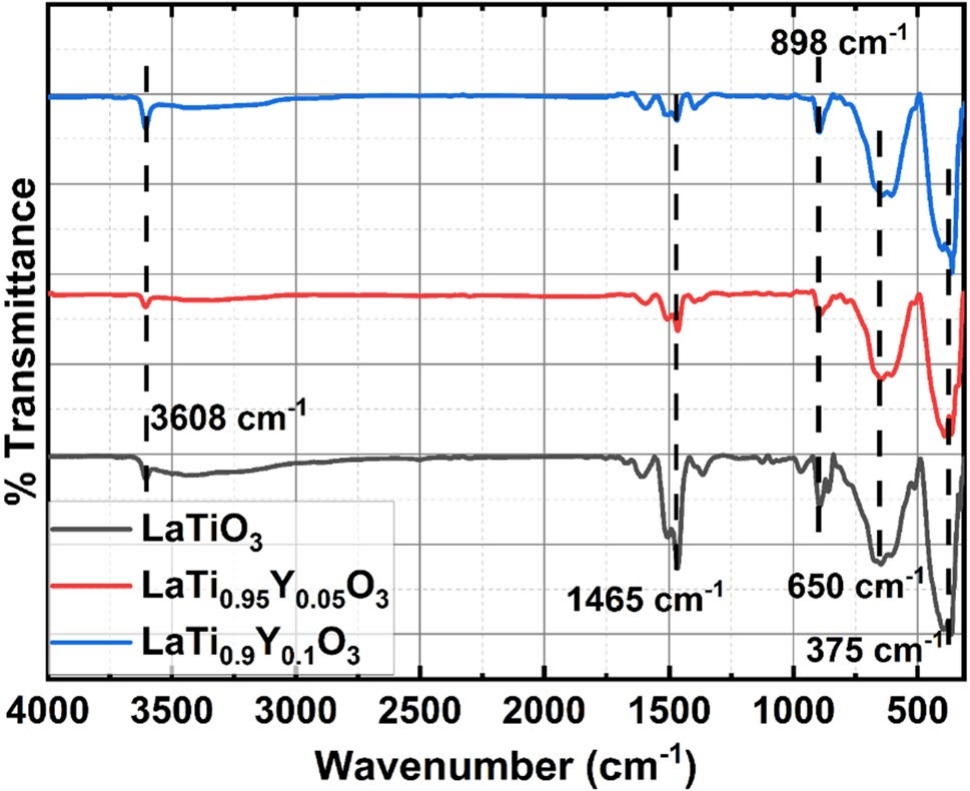
FTIR of LaY_*x*_Ti_1-*x*_O_3_ (*x* = 0, 0.05 and 0.1) from 200 to 4000 cm^−1^

Further, the FT-IR was used to analyse the functional groups of the LYZO nano-catalyst, and Fig. 4 displays the spectrum. From the spectrum, multiple modes of stretching and vibration were seen at various wavelengths. Thus, the O–H, C–O, Ti–O–La, Ti, and La–O stretching vibrations are represented by the sharp IR bands at 3608, 1620, 1465, 950, 898, 650, and 375 cm^−1^, respectively. The acute peak observed at 1465 cm^−1^ corresponds to the interstitial N–O stretching vibrations, whereas the broad band observed in the range of 500–750 cm^−1^ represents the Metal-oxide (M–O=Ti–O) stretching vibrations. The N–H stretching vibration is responsible for the broad peak at about 3115–3608 cm^−1^. The FT-IR peaks are all consistent with LaTiO_3_ characteristics [25, 26].

**Fig. 5:**
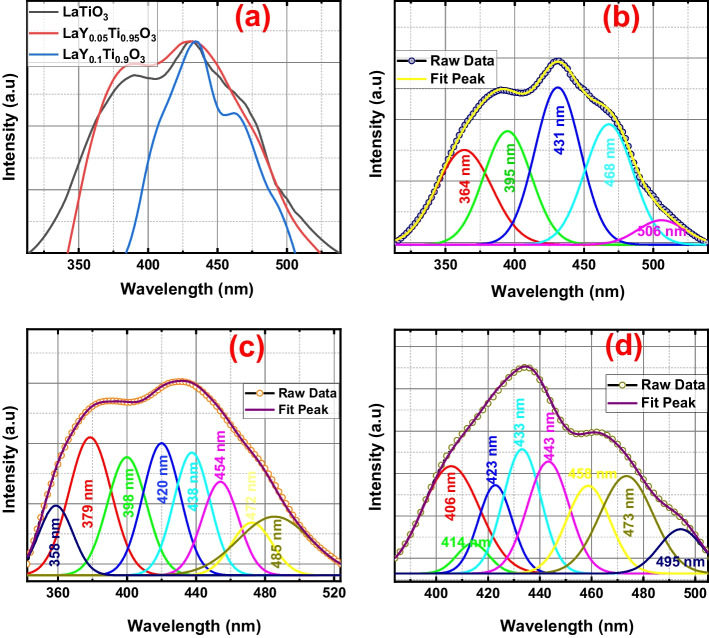
PL of LaY_*x*_Ti_1 − *x*_O_3_ (*x* = 0, 0.05 and 0.1)

A study involving photoluminescence (PL) was conducted to examine the underlying reasons for the enhanced antibacterial efficacy of LYTO in comparison to LTO. The deconvoluted PL spectra of LTO, LY(0.05)TO and LY(0.1)TO is shown in Fig. 5(b) and (c). It is common knowledge that the formation of PL emission spectra may be traced back to the recombination of charge carriers (holes and electrons). Because of this, a greater recombination of photon-induced electron holes will result in a greater PL intensity. The PL spectra of LTO and LYTO nanomaterials are compared in Fig. 5(a). It has been reported that pure LTO emits strongly at a wavelength of approximately 430 nm. However, the diameter of this emission peak is much reduced for the higher doped LTO. This means that the rate of photo-induced electron–hole recombination in hybrids is lower, which makes them more effective at killing bacteria.

**Fig. 6:**
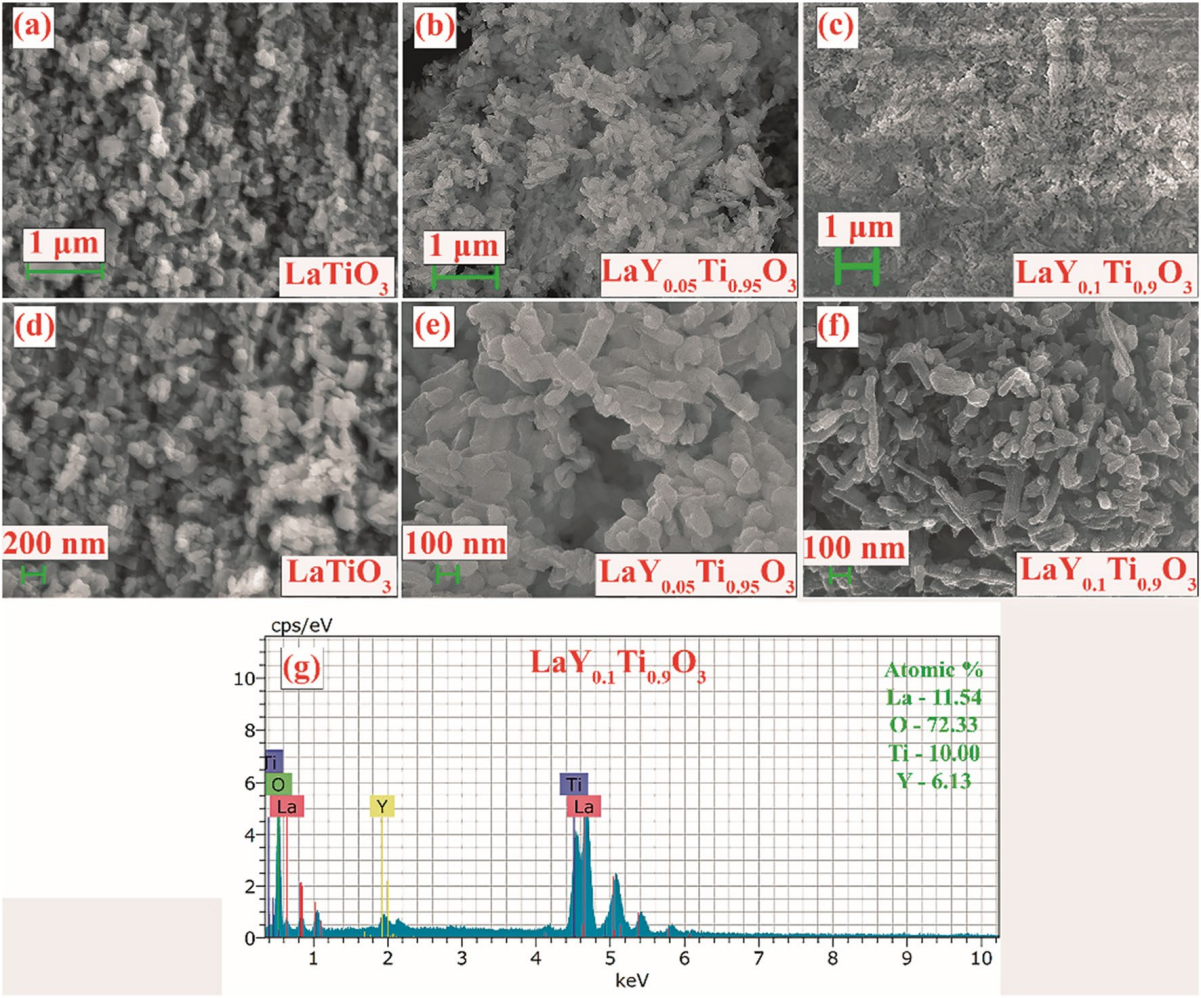
SEM of LaY_*x*_Ti_1 − *x*_O_3_ (*x* = 0, 0.05 and 0.1)

FESEM studies are used to evaluate the nanostructures that have been developed, focusing on their form, size, and distribution. Figure 6a–c show low resolution SEM micrographs of LaY_*x*_Ti_1 − *x*_O_3_ (*x* = 0, 0.05, and 0.1, respectively). Figure 6d–f show high resolution SEM micrographs of LaY_*x*_Ti_1 − *x*_O_3_ (*x* = 0, 0.05, and 0.1, respectively). The FESEM image clearly shows the LTO's irregular spherical nanostructures and the rod-like pellet-shaped Y-LaTiO_3_ nanostructures. The elemental composition of LaY_*x*_Ti_1-*x*_O_3_ (*x* = 0, 0.05, and 0.1) NPs was determined using the EDX technique, and the resulting spectrum is shown in Fig. 6g. The elemental peaks are an accurate representation of the elemental make-up of the proposed nano catalyst. Based on the peaks, the common elements were observed. These were oxygen (atomic percentage = 72.33%), titanium (atomic percentage = 10%), lanthanum (atomic percentage = 11.54%), and yttrium (atomic percentage = 6.13%). These results demonstrated that the LaY_x_Ti_1 − x_O_3_ (x = 0, 0.05, and 0.1) NPs catalyst was successfully manufactured.

**Fig. 7:**
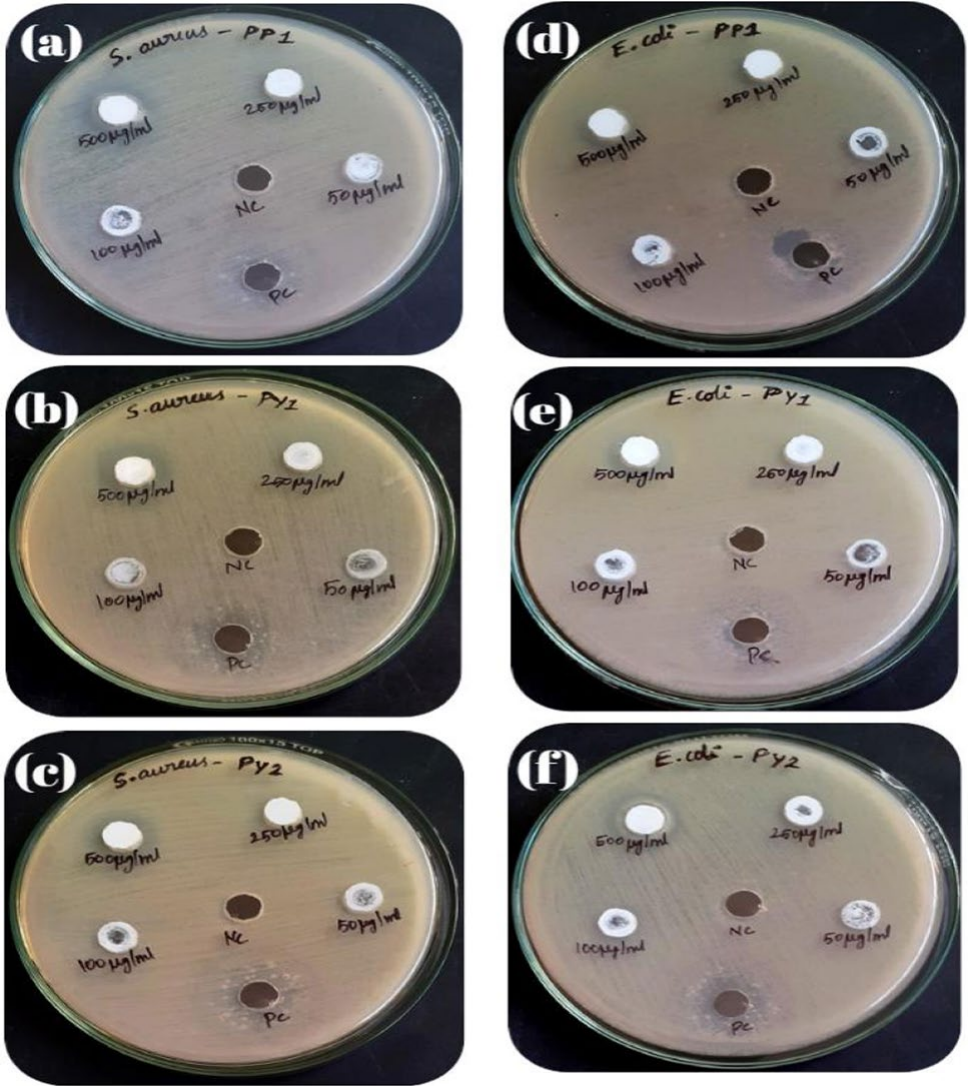
Petri dishes showing zone of inhibition on* S. aureus *due to **a** LTO **b** LY (0.05) TO **c** LY (0.1) TO and on *E. coli* due to **d** LTO **e** LY (0.05) TO **f** LY (0.1) TO

The antibiotics work by a variety of mechanisms, preventing the synthesis of the bacterial cell wall, preventing DNA replication or transcription, or interfering with metabolic pathway or disrupting the cytoplasmic membrane [27]. Antibiotic-resistant bacterial strains are currently becoming more prevalent and emerging. During in vitro experiments, nanomaterials have demonstrated harmful effects against several bacterial species. In this study, an investigation was conducted on the LTO and LYTO nanomaterials with the objective of assessing their efficacy in inhibiting bacterial growth. The diameter of the inhibition zone was employed as a metric for evaluating the extent of bacterial inhibition. Figure 7 shows Petri dishes showing zone of inhibition on* S. aureus* due to (a) LTO (b) LY(0.05) TO (c) LY(0.1) TO and on *E. coli* due to (d) LTO (e) LY(0.05)TO (f) LY(0.1)TO. Table 2 shows how the size of the inhibition zone changes with concentration of the samples. Here concentration of the samples varies from 50 to 500 µg/mL and are tested against *Staphylococcus aureus-*902 (gram positive strain) and *E. coli-*443 (*gram Negative*). All samples of concentration 50 µg/mL and 100 µg/mL show null antibacterial activity against all strains. 250 µg/mL of LY(0.05)TO shows only 40.7% against *Staphylococcus aureus* and LY(0.1)TO shows 31.5% against* E. coli*. Among the three samples LY(0.1)TO of 500 µg/mL shows maximum (87.1%) antibacterial activity (13.5 ± 0.7 mm) compared to positive control (15.5 ± 0.7 mm) against gram positive. It shows maximum (83.3%) activity (11.25 ± 0.35 mm) against gram negative strains also. Even though LTO sample does not have any activities against *E. coli*, it shows significant activity (12.5 ± 0.7 mm) against gram positive (80.1%). The mechanism of action of these nanomaterials on bacterial strains is still under investigation. All three synthesized samples show better activity against gram positive* S. aureus *compared to E coli. Table 3 shows comparison of zone of inhibition against bacterial stains by different metal oxides/perovskite nanoparticles.

**Table 2:** Zone of inhibition (Mean ± SD) obtained by samples against *Staphylococcus aureus* and *E. coli*

Name of the test organism	Name of the test sample	Zone of inhibition (mm) SD ± Mean				
		500 μg/ml	250 μg/ml	100 μg/ml	50 μg/ml	PC
LTO	*Staphylococcus aureus*	12.5 ± 0.7	0	0	0	15.5 ± 0.7
LY(0.05)TO	*Staphylococcus aureus*	11.5 ± 0.7	5.5 ± 0.7	0	0	13.5 ± 0.7
LY(0.1)TO	*Staphylococcus aureus*	13.5 ± 0.7	0	0	0	15.5 ± 0.7
LTO	*E. coli*	0	0	0	0	10.5 ± 0.7
LY(0.05)TO	*E. coli*	9.5 ± 0.7	0	0	0	13.5 ± 0.7
LY(0.1)TO	*E. coli*	11.25 ± 0.35	4.25 ± 0.35	0	0	13.5 ± 0.7

**Table 3:** Comparison of zone of inhibition against bacterial stains by different metal oxides/ perovskite nanoparticles

Name of the test materials	Name of the test organism	Zone of inhibition (mm) SD ± Mean	Concentration	References
Iron nano particle	*S. aureus*	13.5	10 μg/mL	[35]
		13.8	50 μg/mL	
	*E. coli*	12.2	10 μg/mL	
		14	50 μg/mL	
ZnO/4Azeolite	*S. aureus*	6.21 ± 0.02	4 mg/mL	[36]
TiO_2_/4Azeolite		7.58 ± 0.6	4 mg/mL	
TiO_2_ and ZnO/4Azeolite		9.22 ± 0.02	2 mg/mL	
ZnO/4Azeolite	*E. coli*	6.86 ± 0.03	2 mg/mL	
TiO_2_/4Azeolite		9.13 ± 0.03	2 mg/mL	
TiO_2_ and ZnO/4Azeolite		10.73 ± 0.04	1 mg/mL	
Pure ZnTiO_3_	*S. aureus*	9	0.01 ml fixation	[37]
ZnTiO_3_: Ag (0.03%)		16		
LaCo_0.4_Fe_0.6_O_3_	*S. aureus*	16	200 ppm	[38]
	Gram-negative Pseudomonas aeruginosa	18		

Each nanoparticle has a unique mechanism for its antibacterial action. The antibacterial mechanism is not fully understood for any type of nanoparticle. While some mechanisms are related to the physical structure of the nanoparticles, others are related to the improved release of antibacterial metal ions from nanoparticle surfaces. Among the most important factors influencing antibacterial activity are structure, particle size, shape, and zeta potential. [4, 28]. The LTO nanoparticles can penetrate through the bacterial walls and cause further damage to cell due its low crystalline size.

Among the four concentrations, 500 µg/mL had the highest Zone of inhibition against both bacterial stains. According to the current investigation, these nanoparticles have an effective inhibitory impact on* S. aureus *at 500 µg/mL, but they do not significantly affect *E. coli*. This finding is consistent with a study conducted by Tran et al. [29]. Premanathan and colleagues also found in a related investigation that ZnO nanoparticles had significantly more antibacterial action against* S. aureus *than *E. coli* [30]. The antibacterial activity of gram-positive and gram-negative bacteria can differ due to differences in their cell wall composition and structure [29]. 90% of the peptidoglycan layer in the structure of gram-positive bacteria's single-layered cell walls has a low lipid content (1–4%), which allows the entry of bioactive material into the cells. Gram negative bacteria have more complex cell walls that are made up of three layers: an inner layer of peptidoglycan with a lipid content of 11–12%, an outer layer of lipoprotein, and a middle layer of lipopolysaccharide that serves as a barrier to the entry of antibacterial bioactive ingredients. The outer bacterial membrane of *E. coli*, which has a net negative charge because of the lipopolysaccharide and membrane, is likely to have experienced a severe electrostatic repulsion with the Se nanoparticles, as Tran reports [29]. Yttrium addition results in a reduction in particle size, as demonstrated by XRD studies, and it was discovered that antibacterial activity of nanoparticles increased with decreasing particle size; the increased bioactivity of smaller particles is likely due to the higher surface area to volume ratio [31].

Antioxidants are classes of substances that neutralize reactive oxygen species (ROS) and free radicals. It is essential in food production and processing as it can enrich and preserve food. Here, 1-diphenyl-2-picryl hydrazyl (DPPH) has been employed as a reagent to examine the free radical-scavenging capacities of several substances [32, 33]. When scavenged, the stable free radical DPPH changes from pink to yellow. In the presence of a hydrogen-donating antioxidant, alcoholic DPPH solution is reduced to non-radical form DPPH–H [33]. Table 4 shows the scavenging activity of the samples with different concentration. The absorbance reduces as sample concentration rises, and percentage radical scavenging activity rises as sample concentration rises as well. The concentration of the sample that can scavenge 50% of DPPH radicals (IC50 value) were found to be 352.33 µg/mL, 458.055 µg/mL, 440.163 µg/mL (Calculated using ED 50 PLUS V1.0 Software) for LaY_*x*_Ti_1 − *x*_O_3_ (*x* = 0, 0.05 and 0.1) respectively. IC50 values of different nano materials were compared in Table 5. Substances with antioxidant action delay or inhibit substrate oxidation [34]. By chelating transition metals or giving up electrons, antioxidants neutralize free radicals. Unstable chemicals called free radicals damage cell DNA. They are produced by normal metabolic processes or exogenous sources. Free radicals can produce oxidative stress, which increases disease risk, including cancer [34]. So, by analyzing antioxidant and antibacterial properties of the synthesized samples will reveals the potential of our materials as a practical antioxidant and a good antibacterial nano material. The significant antioxidant property is a consequence of the increased surface area even though, the mechanism behind the reactivity towards DPPH radical needs to be investigated further, the synthesized nanoparticles can be employed well in therapeutic applications.

**Table 4:** Variation of scavenging activity with different concentrations of LYTO

Sample	Concentrations (µg/mL)	Absorbance	Percentage of inhibition
Control		0.6654	0.00
LTO	12.5	0.5894	11.4217
	25	0.5677	14.6829
	50	0.5499	17.35798
	100	0.4925	25.98437
	200	0.4525	31.99579
LY(0.05)TO	12.5	0.5968	10.30959
	25	0.5759	13.45056
	50	0.5321	20.03306
	100	0.5019	24.57169
	200	0.4879	26.67568
LY(0.1)TO	12.5	0.5935	10.80553
	25	0.5874	11.72227
	50	0.5525	16.96724
	100	0.5127	22.9486
	200	0.4848	27.14157

**Table 5:** Comparison of antioxidant properties of different metal oxides

Sample	IC50 values (µg/mL)	References
SnO_2_	206.278	[39]
Sn_0.93_Mn_0.07_O_2_	381.892	
CuO	131.592	[40]
Cu_0.93_Co_0.07_O	189.058	
Cu_0.93_Ni_0.07_O	158.146	[18]

## Conclusion

LYTO (LaY_*x*_Ti_1 − *x*_O_3_) NPs were synthesised by auto combustion method and have been characterized by XRD, UV, FTIR, PL, FESEM and EDX techniques. XRD reveals excellent crystalline structure with tetragonal crystal system and calculated crystalline size are 19, 13 and 12 nm for *x* = 0, 0.05 and 0.1. The FESEM micrographs exhibited irregular spherical nanostructures and the rod-like pellet-shaped Y-LaTiO_3_ nanostructures and elements are confirmed with the EDAX spectrum. Further, PL indicates that the photo-induced electron–hole recombination rate in doped LYTO is reduced and hence it promotes their increased antibacterial activity. The antibacterial activity was studied against, *E. coli*, and the data showed that the inhibitory activity of NPs is concentration dependent. 10% Yttrium doped LaTiO_3_ has maximum and efficient antibacterial activity to both gram-positive *Staphylococcus aureus* and gram-negative *E. coli.* Free radical scavenging activity of all samples shows significant IC50 values. The material with general formula LaY_*x*_Ti_1 − *x*_O_3_ has unique characteristics which is a promising candidate for the application in biomedical field.

## Data Availability

Data will be made available on request.
